# Supplementary material for: Evidence needs and community involvement in policy decisions for vaccine-preventable diarrhoeal infections among children under the age of five years: Stakeholder engagement in Ethiopia, Kenya, and Malawi
Source: PLOS Glob Public Health. 2025 Dec 1;5(12):e0004775. doi: 10.1371/journal.pgph.0004775 (PMC12668512; doi:10.1371/journal.pgph.0004775)
Supplement: S1 Text — (PDF) [file pgph.0004775.s001.pdf]

S1_Text

Topic Guides

Topic guide for Institutional Stakeholders

Recorder set and ready:

1. **Introduction**

1.1 Please briefly explain the primary mission of your organisation, your role/title and involvement in issues (if any) related gastrointestinal infections among children under the age of five years in this country.

1.2 How many years have you been in this organisation?

	**2. Involvement in policymaking (question to consider)**
2.1	How important do you believe it is for stakeholders (interested or concerned parties) to be involved in the policy-making process? Why do you think so?
2.2	In your opinion, what types of stakeholders should be involved in policy making for vaccine-preventable gastrointestinal infections among under five children in your country?
2.3	Have you ever participated in a policy-making process for vaccine-preventable gastrointestinal infections among under five children in your country?<br>If 'Yes', what was your role and highlight of your experience? What was the biggest challenge?
2.4	How would you prefer to be involved in policy making for vaccine-preventable gastrointestinal infections among children under the age of five years?
2.5	How can policymakers best communicate with stakeholders (interested or concerned parties) during the policy-making process in this country?
2.6	Do you feel that your input in policy making is valued and considered?<br>Please explain your choice with an example of how it was/wasn't.
2.7	What suggestions do you have to improve stakeholder involvement in policy making?
3.3	Have you successfully advocated for policy changes in the past? If so, could you provide an example?
	**3. Engagement**
3.1	How involved are you or is your organization in health policy discussions or advocacy efforts for gastrointestinal infections among children aged under five in your country?
3.2	What barriers or challenges would you anticipate in promoting or supporting health policy change for gastrointestinal infections in under five children in your country? Please explain further about your choices.
	**4. Data and information needs**
4.1	To change or sustain a health policy for vaccine-preventable gastrointestinal infections among children under the age of five years old, what type of data and information do you think would be required to influence decision making?
4.2	Where do you think this data and information should come from to trust it?
4.3	Who do you think is legit to provide this data and information? Why is it so?
4.4	When should this data and information be available or communicated to stakeholders?
4.5	What approached would you suggest in promoting the use of data/evidence in policy decision making?

Please, provide and additional comment.

<u>Topic guide for focus group discussion with Community Participants</u>

Introduction

- To define gastrointestinal infections and how vaccines are used to control some of these infections
- To provide an example of the rotavirus vaccine as an existing policy in the country's Expanded Programme on Immunization (EPI)
- To briefly highlight how policies are made in a country and need for involving relevant stakeholders

	**1. Interest, priority, awareness, and understanding**
1.1	What are the top health concerns or priorities among under five children in your community?
1.2	How important are gastrointestinal infections among children to you personally?
1.3	What existing health policies are you aware of that are related to vaccines for gastrointestinal infections among children under the age of five years?
1.4	How well do you understand the current health policy and its implications?
1.5	What do you think about introducing new vaccines for other gastrointestinal infections?
1.6	What information would you need to consider before the introduction of these new vaccines?
	**2. Personal experience**
2.1	Have your children or other children you know been directly affected by gastrointestinal infections?
2.2	What challenges have you faced in accessing healthcare services related to gastrointestinal infections?
	**3. Decision-making power and influence**
3.1	Do you feel that you can influence the decisions made by authorities/government on choices of vaccines for gastrointestinal infections among children under the age of five years old? (to probe on their reasons behind the option) – The idea is to find out if they think they can influence policy change.
3.2	Are there any channels or platforms where you can voice your concerns and suggestions about issues affecting health in this community? (To probe what strategies are available)
	**4. Engagement and participation**
4.1	Have you participated in any public consultations or community forums related to health policy changes?
4.2	How likely are you to actively engage in discussions and activities related to gastrointestinal infections? Why?
4.3	Are there any barriers preventing your engagement in health policy discussions or initiatives?
	**5. Community resources and collaboration**
5.1	Are there community organizations or groups working on health-related issues, including vaccine-preventable gastrointestinal infections?
5.2	Have you collaborated with other community members or organizations to address health concerns or advocate for policy changes?
	**6. Desired outcomes**
6.1	What specific outcomes or improvements would you like to see in the health policy related to vaccine-preventable gastrointestinal infections?
	**7. Feedback and suggestions**
7.1	Do you have any specific feedback or suggestions for improving the current health policy related to vaccine-preventable gastrointestinal infections?
7.2	What additional support or resources do you think would be helpful in addressing vaccine preventable gastrointestinal infections?
