# Supplementary material for: Evidence needs and community involvement in policy decisions for vaccine-preventable diarrhoeal infections among children under the age of five years: Stakeholder engagement in Ethiopia, Kenya, and Malawi
Source: PLOS Glob Public Health. 2025 Dec 1;5(12):e0004775. doi: 10.1371/journal.pgph.0004775 (PMC12668512; doi:10.1371/journal.pgph.0004775)
Supplement: Attachment — Submitted filename: Responding to reviewers.docx [file pgph.0004775.s006.docx]

**Responding to reviewers’ comments**

Academic Editor, PLOS Global Public Health

Dear Dr. Rajiv Sarkar,

**Re: PGPH-D-25-01318 - " Evidence needs and community involvement in policy decisions for vaccine-preventable diarrhoeal infections among children under the age of five years: Stakeholder engagement in Ethiopia, Kenya, and Malawi"**

Thank you for the feedback on our manuscript. We greatly appreciate the editorial attention and the opportunity to respond to the reviewers’ comments.

We have carefully revised the manuscript and fully addressed all reviewer comments. Detailed point-by-point responses are provided below. In summary, we have:

- Revised and clarified the methodology section with appropriate subheadings.
- Added relevant literature, including key contributions from icddr,b as recommended.
- Enriched the results, ensuring a clearer balance between the perspectives of institutional stakeholders (ISs) and community participants (CPs).
- Provided greater context for our findings and strengthened the discussion and conclusion accordingly.

We sincerely thank the editorial team and external reviewers for their constructive and thoughtful comments, which have significantly improved the quality and clarity of the manuscript. We believe the revised version will be of strong relevance to readers of PLOS Global Public Health and we look forward to your feedback.

With kind regards,

Chikondi Mwendera

On behalf of all co-authors

|  | **COMMENTS** | **RESPONSE** |
| --- | --- | --- |
|  | ***JOURNAL REQUIREMENTS*** |  |
| 1 | Please provide separate figure files in .tif or .eps format.  For more information about figure files please see our guidelines: <https://journals.plos.org/globalpublichealth/s/figures> <https://journals.plos.org/globalpublichealth/s/figures#loc-file-requirements> | Thank you for pointing this out. We have now saved all figures in the required **TIFF format** and uploaded them as separate files in line with the journal’s submission guidelines. |
| 2 | We have amended your Competing Interest statement to comply with journal style. We kindly ask that you double check the statement and let us know if anything is incorrect. | Thank you for amending the Competing Interest statement to comply with journal style. We have reviewed the revised statement carefully and confirm that it is correct. No further changes are required. |
| 3 | When completing the data availability statement of the submission form, you indicated that you will make your data available on acceptance. We strongly recommend all authors decide on a data sharing plan before acceptance, as the process can be lengthy and hold up publication timelines. Please note that, though access restrictions are acceptable now, your entire data will need to be made freely accessible if your manuscript is accepted for publication. This policy applies to all data except where public deposition would breach compliance with the protocol approved by your research ethics board. If you are unable to adhere to our open data policy, please kindly revise your statement to explain your reasoning and we will seek the editor's input on an exemption. Please be assured that, once you have provided your new statement, the assessment of your exemption will not hold up the peer review process. | Thank you for your guidance regarding the journal’s open data policy. We have carefully considered this requirement considering our study’s ethical obligations.  As noted, our data consist of qualitative interviews and focus group discussions with small numbers of participants in each country. in the case of ministry staff, they represented members of a very small departmental workforce. Due to the small sample sizes and the sensitive nature of views shared, full transcripts cannot be made publicly available. Sharing complete narratives could risk participant identification, which would breach confidentiality agreements and informed consent. Importantly, participants did not consent to public data sharing or deposition in external repositories.  We have therefore revised our Data Availability Statement as follows:  *“Due to the small sample sizes in each country and the sensitive nature of the views shared during interviews and focus group discussions, full transcripts cannot be made publicly available. Sharing complete narratives could risk participant identification, which would breach confidentiality agreements and informed consent. Participants did not consent to public data sharing or deposition in external repositories. To safeguard participant privacy, access to the full data is restricted to the research team. However, de-identified excerpts relevant to and illustrative of the study’s findings are presented within the manuscript.”*  We hope this revised statement provides sufficient clarity and request an exemption from full public data sharing, in accordance with the journal’s policy on studies where ethical considerations limit data accessibility. |
| 4 | If the reviewer comments include a recommendation to cite specific previously published works, please review and evaluate these publications to determine whether they are relevant and should be cited. There is no requirement to cite these works unless the editor has indicated otherwise. | We thank the reviewers for their recommendations regarding relevant literature. We carefully reviewed the suggested publications and evaluated their relevance to our study. Where appropriate, we have incorporated these references into the Introduction and Discussion to strengthen the manuscript and situate our findings within the broader evidence base. |
| 5 | Reviewer 1 has provided several valuable suggestions, both within the manuscript and in her written comments, including recommendation for referencing relevant work on diarrheal diseases conducted by ICDDR, B. Kindly consider citing appropriate publications from Bangladesh and other LMICs (outside of Africa), as applicable. | We thank Reviewer 1 for her constructive and detailed comments, which have significantly strengthened the manuscript. We have carefully addressed all suggestions, including those highlighted directly within the manuscript. In particular, we have incorporated references to relevant work from icddr,b in Bangladesh, acknowledging its pioneering role in diarrhoeal disease research and vaccine development. |
|  | ***REVIEWER 1*** |  |
| 1 | The methodology section should be clearer and rewritten. The literature review should be more in-depth. International Centre for Diarrhoeal Disease Research, Bangladesh (icddr,b) has been doing diarrhoeal diseases research for over 6 decades and there is no mention or reference to the research work of this organization. If the result section is enriched with further edits, the Conclusion will take a new shape. In terms of data availability - the guidelines are there. | We thank the reviewer for these critical and constructive comments. We have carefully revised the manuscript in line with this feedback, including strengthening the methodology, expanding the literature review, integrating relevant references to icddr,b, enriching the results, and reshaping the conclusion. We also updated the data availability statement in line with journal guidelines. Specific details on how these have been addressed are presented in the points below. |
| 2 | so, this is qualitative research by design? Please mention this here. | We thank the reviewer for this observation. We have clarified in the *Abstract –* (Page 1, line 31) that this is a qualitative study. We have also revised in the Abstract that the study employed a qualitative design. |
| 3 | In the introduction there is need a bit robust literature review. Bangladesh is a success case in this field after combating for decades in this field. Please mention Bangladesh which will enrich the literature review. | We thank the reviewer for this valuable suggestion. The *Introduction* has been revised to strengthen the literature review and to acknowledge Bangladesh as a success case in diarrhoeal disease research and control. Specifically, we now highlight the pioneering role of icddr,b in developing oral rehydration solution (ORS) and its continued leadership in vaccine research, which have shaped global diarrhoeal disease policies and provide important lessons for LMIC contexts (Pages 2-3, lines 73-78). |
| 4 | Methods are the technics that you employ to gather the data under which the IDIs, FGDs and institutional stakeholders should come. Did you mean methodology? Also, methodology section should include sample size, how the sample was selected and how the study site was identified. You provided the number of each informant in the abstract, but not in the main paper. This should be included here too. Please re-write the methodology section again to give a clearer idea of the research process | We thank the reviewer for this valuable feedback. In response, we have restructured and expanded the section, now presented under the heading *Methodology* (pages 3-7). Subsections have been added to provide greater clarity on the research process under Study design, participants, setting and recruitment, Data collection, and Data management and analysis. WE have also ensured that participants numbers are clearly reported in this section. |
| 5 | Since it is acceptance of new vaccine, why no interviews with community family members (care givers of babies and children). Please give a justification. One way is to recommend future research to explore these issues with parents | We thank the reviewer for raising this important point. This study was primarily focused on exploring participants’ involvement in vaccine policy processes, with institutional stakeholders reflecting on evidence use and decision-making, and community representatives discussing their perceived influence through CEI structures. We did not therefore include caregivers in this study, as the aim was not to assess household-level knowledge, attitudes, or acceptance of vaccines.  However, we note that other studies within the GHRG-GI are specifically examining caregivers’ perspectives on the introduction of new vaccines, including knowledge, attitudes, and practices related to childhood diarrhoeal diseases. Findings from those studies will be published separately.  In response to this comment, we have also revised the statement on future research (in the Strength and Limitation section) to directly explore these issues with caregivers, as their perspectives are essential to understanding vaccine acceptance. |
| 6 | How did you select this? Please mention | Thank you for your comment. The information on site selection has been included under ‘Study Sites’ in the methodology section. We have clarified that sites were selected based on challenges related to access to infrastructure and resources, and that the selection was guided by our collaborating national partners in each country |
| 7 | So, the FGDs and IDIs were also carried out in these areas, I assume. Therefore, this is the piece that should come under study site selection | This has been revised and included under ‘Study Sites’ in the methodology |
| 8 | In terms of what? | ‘Study relevance’ as included in this sentence has been deleted to avoid confusing the readers. |
| 9 | This is contradictory – a structured guideline cannot have open ended questions – the structured question may lead you to some of the open-ended question to some extent. Please reconsider how you want to present it | We thank the reviewer for pointing out this inconsistency. In the revised manuscript, we have removed the reference to “open-ended questions” and clarified that data collection was guided by a structured question guide. The guides included prompts that allowed participants to expand on their responses, but we now consistently describe them as topic guides to avoid confusion (Methods – Data Collection, page 6, line 146). |
| 10 | Why in English? Why could not you hire local researchers? Please give justification. | Thank you for this important query. We have clarified these points in the revised manuscript (see *Methods, Data Collection*, Page 6, lines 149-152).  The institutional stakeholders interviewed for this study were national-level actors drawn from ministries of health, research institutions, and partner organisations. All of them were fluent in English and routinely conducted their professional work in English. As such, interviews were comfortably conducted in English without the need for translation. For this reason, the first author, who is experienced in qualitative interviewing, facilitated these interviews directly. |
| 11 | Please provide explanation why it was necessary to carry out the interview virtually sometimes | We agree that in-person interviewing is preferable, and this was our first choice. The first author travelled to each country to conduct the interviews in person; however, several key stakeholders were unavailable due to competing professional commitments. At their request, interviews were therefore conducted virtually as a second option to accommodate their participation. This flexibility ensured inclusion of otherwise inaccessible participants without compromising the quality of the data. (Methods – Data Collection, page 6, line 152-155).  However, the FGDs were conducted in local languages, hence they were facilitated by local research team members in each country. |
| 12 | Please specify where did the FGDs carried out and who and how many were there in each FGD. | Thank you for this helpful comment. We have now clarified in the Methods-Data Collection (page 6, lines 153-158) that FGDs were conducted in the usual CEI meeting environments in each country, which allowed participants to feel comfortable and familiar with the setting. Specifically, the Ethiopian FGD was held at Kirkos Health Centre (Addis Ababa), the Kenyan FGD at Mukuru Health Centre (Nairobi), and the Malawian FGD at Bangwe Health Centre (Blantyre). The FGDs were facilitated by trained social scientists from the GHRG-GI country teams and co-authors: SW in Ethiopia, RO in Kenya, and MP in Malawi. |
| 13 | See these are the issues that are better explored through experiences of caregivers. Please give a justification why caregivers were not included in the research. OR, If it did, I did not clearly understand that. Please make it clear for the readers if you had included them | Thank you for this important point. We have clarified in the revised manuscript (Methods – Data Collection, page 6, lines 159-160) that this study did not seek to explore caregivers’ knowledge of diarrhoeal diseases or vaccines. Instead, our focus was on institutional stakeholders’ perspectives on evidence use in vaccine policymaking, and community representatives’ perceptions of their influence in decision-making through CEI structures. While caregivers were not included as participants in this study, parallel country-specific studies within the GHRG-GI are investigating caregivers’ knowledge, attitudes, and practices regarding childhood diarrhoeal diseases and vaccines, and will be published separately. |
| 15 | Most of the result section is expanded with lots of quotes instead of presenting the result revealed through triangulation (among countries and types of informants). While quotes are good, but it is usually used as supporting evidence. You do not present the result section with lots of quotes. Also, you do not need to give quotes from all three countries while supporting an statement. | We thank the reviewer for this constructive observation. We have revised the *Results* section to improve balance between narrative and supporting quotes. In particular, Themes 1, 2, 3, and 4 have been substantially revised: the number of quotes has been reduced, and the analysis is now presented more concisely to reflect triangulation across both institutional stakeholders (ISs) and community participants (CPs) where necessary. Where quotes are included, they are used selectively to illustrate key points rather than as the primary means of presenting findings. We also streamlined country-level quotations, avoiding repetition unless differences between contexts were important to highlight. These revisions provide a clearer synthesis of the results while retaining illustrative voices from participants. |
| 16 | This should go to the methodology section. Also, wasn’t this part of identifying/selecting the informants through “through a participatory, context-sensitive process”? Please make sure how you want to present this – as sample identification or as data collection. | Thank you for this helpful clarification. We have now moved this description to the Methodology section under Participants, recruitment and setting, pages 4-5 where we describe it as part of the process of sample identification and recruitment. To avoid overlap with the Data Collection subsection, we clearly distinguish between how participants were identified (through a participatory, context-sensitive process involving community nomination and selection) and how the FGDs and IDIs were subsequently conducted. In addition, Table 1 has been revised to present participant characteristics in aggregated form, thereby preserving confidentiality while maintaining transparency. |
| 17 | Is it possible to simplify the table? Or you may have a short paragraph on this as result section and then provide the table as a supplement? | We thank the reviewer for this suggestion. To minimise the risk of identifying participants, particularly given the relatively small number of national-level institutional stakeholders in each country, we have simplified Table 1 in the revised manuscript. Participant characteristics are now presented in aggregated form: institutional stakeholders are grouped as government, research, or partner organisation representatives, and community participants are described at the focus group level. This approach maintains transparency regarding the diversity of participants while upholding ethical standards of confidentiality. The change is reflected in Table 1 and explained in the Methods (Participants subsection, page 5). |
| 18 | This also should go under methodology section | This has been moved to the methods section as suggested. |
| 19 | This section could be made more succinct presenting validating the findings from the two sets of data (IDI and FGD) instead of using too many quotes | We thank the reviewer for this useful suggestion. We have revised the *Results* section, particularly Theme 1, to make it more succinct. The number of quotes has been reduced, and the narrative has been streamlined to integrate perspectives from both institutional stakeholders and community participants. This revision allows findings from IDIs and FGDs to be presented in a more balanced and coherent manner, while retaining illustrative quotes that best support the key themes. |
| 20 | However, you did not explore this through your research. Also why suddenly this statement is here? | We thank the reviewer for this observation. In revising the manuscript, we have deleted the sentence in question to avoid introducing issues that were not directly explored in our research. The focus of this section is now streamlined to emphasise the importance of generating population-based outputs using accurate and consistent metrics to guide planning for the introduction of new vaccines. This ensures alignment with the study findings and maintains coherence in the narrative. |
| 21 | Here you should be talking about Bangladesh which is an example of such in many instances especially diarrhoeal diseases | Thank you for this suggestion. We have revised the Discussion (page 16-17, lines 422-429 and lines 435-439) to reference key contributions from icddr, b, including work on rotavirus vaccine trials, and oral cholera vaccine feasibility studies. These examples strengthen our argument by illustrating how locally generated multidisciplinary and programmatic evidence has informed national diarrhoeal disease control policies in a resource-limited setting. |
| 22 | ICDDR,B (international Centre for Diarrhoeal Diseases and Reseach, Bangladesh) should be mentioned here which has been doing research for over 60 decades in collaboration with various international institutions and organizations | We thank the reviewer for this valuable suggestion. In the revised manuscript, we have now acknowledged the historical importance of icddr,b and its contribution in shaping global policies on diarrhoeal disease control. Specifically, we highlight icddr,b’s pioneering development of oral rehydration solution (ORS) in the 1960s, which stands as a landmark example of how long-term investment in diarrhoeal disease research within an LMIC can generate evidence with direct and lasting global policy relevance (Page 17, lines 435-439) |
| 23 | You did not talk much about side effects in the result – please try to incorporate those issues in the discussion section which you have discussed in the result section | We thank the reviewer for this important observation. In the revised manuscript, we have clarified that community participants (CPs) raised the need for clear and accessible information on vaccine characteristics, including side effects, safety, and mode of administration, as critical factors for community acceptance of new vaccines. In the *Discussion* (Page 18, lines 446-447), we are only highlighting that transparent communication on these vaccine characteristics is essential to build trust and should therefore form part of future vaccine introduction strategies. |
| 24 | Please do a more deep literature review. There are lots of literatures on both of these issues and many more related to diarrhoeal diseases | Thank you — we agree that the literature of community perspectives on diarrhoea management and vaccine acceptance acceptability is large and important. In our case we have recommended that contextual specific empirical studies need to be conducted amid preparation of Shigella or ETEC vaccine introduction once they become available. |
| 25 | Once the above comments will be integrated, I understand the conclusion will take a different shape | The Conclusion has been revised to reflect the integrated changes across the manuscript, including a more balanced synthesis of findings from institutional stakeholders and community participants, acknowledgement of evidence-related challenges, and clearer recommendations for future research and policy. The revised Conclusion now emphasises the four key evidence domains, highlights the importance of transparent communication with communities, and situates the study within the broader context of strengthening local research capacity and long-term investment, with reference to examples such as icddr,b. |
|  | ***REVIEWER 2*** |  |
| 1 | Can you expand more on the study design, why phenomenological? | We agree this needs clarification. We have expanded the explanation of the phenomenological design, highlighting that it enabled us to explore participants lived experiences, perceptions, and meanings related to their involvement in vaccine policy processes.  See Study design subsection (Page 3, lines 95-98). |
| 2 | The selection of the settings of the FGD's is not optimal, urban areas like AA and Nairobi have significantly higher access to immunization services than their rural counterparts, also less likely to suffer from diarrheal diseases and its complications. This should be highlighted as a limitation. | We acknowledge this point and have now justified the selection of Addis Ababa, Nairobi, and Blantyre as study sites (due to existing CEI groups and feasibility within the GHRG-GI platform). We also explicitly acknowledge in the Limitations section that urban settings may not reflect rural experiences, where disease burden and immunisation access differ.  Strengths and limitations (Page 19, lines 480-482). |
| 3 | Under emerging themes, the first theme is "policy involvement and evidence-related challenges" and then its titled "policy involvement", which one is it? | Thank you for pointing out this inconsistency. We have revised the text so that the theme is consistently titled **“Policy involvement and evidence-related challenges.”**  See Results, Emerging Themes (Page 8, line 199). |
|  | The quotes of theme 1 are focused on the roles of the participants in immunization programs nothing significantly related to "evidence related challenges were captured" except issues raised by a respondent in line number 196-198. | We appreciate this observation. We have revised the *Results* presentation under Theme 1 to integrate both “policy involvement” and “evidence-related challenges.” Additional illustrative quote has been included to highlight barriers such as quality timeliness and completeness of surveillance data.  See *Results, Theme 1* (Page 9, lines 218-228). |
|  | On theme 3...are there any FGD participants that had ideas on who would be the trusted sources of evidence for the community? | Thank you for this valuable point. We reviewed the FGD transcripts and found that community participants identified **health workers, community leaders, and religious leaders** as their most trusted sources of health and vaccine information. We have now integrated these insights into Theme 3 (trusted sources of evidence) instead of only presenting them in the communication strategies section. Where community participants did not specify “evidence sources” in technical terms, we clarify this distinction. See Results, Theme 3 (Pages 12-13, lines 308-326). |
